# Occupational ALARA Planning for Reactor Pressure Vessel Dismantling at Kori Unit 1

**DOI:** 10.3390/ijerph17155346

**Published:** 2020-07-24

**Authors:** Juyoul Kim, Batbuyan Tseren

**Affiliations:** Department of NPP Engineering, KEPCO International Nuclear Graduate School, 658-91 Haemaji-ro, Seosaeng-myeon, Ulju-gun, Ulsan 45014, Korea; babu4811@gmail.com

**Keywords:** occupational dose, ALARA, decommissioning, nuclear power plant

## Abstract

Assessing workers’ safety and health during the decommissioning of nuclear power plants (NPPs) is an important procedure in terms of occupational radiation exposure (ORE). Optimizing the radiation exposure through the “As Low As Reasonably Achievable (ALARA)” principle is a very important procedure in the phase of nuclear decommissioning. Using the VISIPLAN 3D ALARA planning tool, this study aimed at assessing the radiological doses to workers during the dismantling of the reactor pressure vessel (RPV) at Kori NPP unit 1. Fragmentation and segmentation cutting processes were applied to cut the primary component. Using a simulation function in VISIPLAN, the external exposure doses were calculated for each work operation. Fragmentation involved 18 operations, whereas segmentation comprised 32 operations for each fragment. Six operations were additionally performed for both hot and cold legs of the RPV. The operations were conducted based on the radioactive waste drum’s dimensions. The results in this study indicated that the collective doses decreased as the components were cut into smaller segments. The fragmentation process showed a relatively higher collective dose compared to the segmentation operation. The active part of the RPV significantly contributed to the exposure dose and thus the shielding of workers and reduced working hours need to be considered. It was found that ^60^Co contained in the stainless steel of the reactor vessel greatly contributed to the dose as an activation material. The sensitivity analysis, which was conducted for different cutting methods, showed that laser cutting took a much longer time than plasma cutting and contributed higher doses to the workers. This study will be helpful in carrying out the occupational safety and health management of decommissioning workers at Kori NPP unit 1 in the near future.

## 1. Introduction

Kori unit 1, a two-loop Westinghouse pressurized light water reactor with an output of 587 MWe, was the first Korean commercial nuclear power plant (NPP) and started its operation in April, 1978 and was permanently shut down for decommissioning in June, 2017. The national strategy for the decommissioning of Kori unit 1 was an immediate decommissioning (DECOM) and the end state of the NPP site was determined as brownfield land, where the site release criterion would be 0.1 mSv/yr. The nuclear decommissioning of Kori unit 1 was scheduled to last for at least 15 years; the cooling of spent nuclear fuel in 2017–2022, decontamination and dismantling in 2022–2028, and site restoration by 2032 [1,2]. During the decommissioning phase, several activities, including the preparation of a final decommissioning plan (FDP) which should be submitted to the regulator within 5 years after permanent shutdown, i.e., by June, 2022, public hearings with residents near Kori NPP unit 1 for 1 year, the completion of spent nuclear fuel transfer by 2025, the decontamination and dismantling of structures, systems and components (SSCs) of Kori unit 1, the treatment and conditioning of radioactive wastes and the storage and the final disposal of radioactive wastes, have to be undertaken with proper steps in order to achieve the desired end state within the planned time schedule. However, the activities of the dismantling and segmentation of the activated reactor pressure vessel (RPV), RPV internals and contaminated steam generators and pressurizers pose a serious radiation exposure risk to the decommissioning workers. One of the most challenging tasks during the decommissioning of Kori unit 1 was considered to be the removal of highly radioactive internal components of the RPV [3]. Boric acid was used in Kori unit 1 coolant and hence the use of stainless steel was required for the protection of reactor internals inside of a carbon steel reactor vessel. Cobalt and other metals, such as nickel, were some of the impurities used in the stainless steel and were ultimately activated and contributed to high radionuclide concentrations in the RPV and RPV internals. Ample knowledge of the “As Low as Reasonably Achievable” (ALARA) principle of radiation protection is inevitable to optimize the exposure of radiation workers. ALARA means making every reasonable effort to keep the exposure of radiation workers as far below the limits as possible, consistent with the purpose for which the license activity was undertaken in relation to benefits to the workers’ health and safety, and other socio-economic considerations. Dose planning and estimation were considered as vital phases of the ALARA principle implementation, thus the selection of proper technologies of dismantling equipment that allow for reducing the personnel’s collective dose was highly recommended. The three principles used in ALARA for reducing external exposure are time, distance and shielding. Various types of software that take into account the occupational radiation exposure have been developed to plan the dismantling activities [4,5,6]. In this paper, the modeling results of radiation doses to workers during the dismantling of a reactor pressure vessel at Kori unit 1 was conducted using VISIPLAN computer code [7,8]. The computer code “VISIPLAN 3D ALARA planning tool”, developed by the SCK-CEN Laboratory in Belgium, has been widely used to solve radiation protection problems resulting from exposure to direct radiation, such as the handling of fiber-reinforced concrete containers with conditioned radioactive waste [9,10,11,12,13].

## 2. Materials and Methods

**Figure 1:**
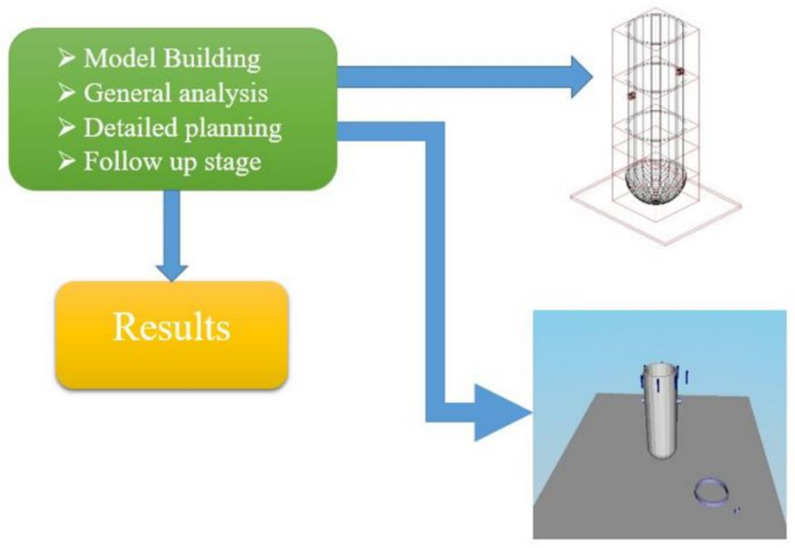
The main stages in the methodology of VISIPLAN.

The VISIPLAN 3D ALARA planning tool is a new calculation tool developed to facilitate the planning of the work based on 3D geometrical, material and radiological information. The software considers dose assessments for external exposure to gamma radiation. The dose calculations are based on a point-kernel method with a build-up correction, whereby each small source is called a kernel, and the process of integration, where the contribution to the dose of each point is added up, is called “point kernel” integration. The VISIPLAN methodology consists of four steps: model building, general analysis, detailed planning and follow-up, as shown in Figure 1.

The model building stage, which is the first step in the analysis, is the characterization of the site or work area. The geometrical and material information required can be derived from technical drawings or survey techniques. Once the model is defined, the general analysis stage follows and involves the calculation of dose maps of the working areas. The dose rates can be displayed as contours or colorful patterns on grids perpendicular to the x, y and z axes of the model. The tools available for the detailed planning phase involve a trajectory calculation and a scenario building tool. The trajectory contains information involving the task description, the location and the duration of the sequential tasks to be performed. The graphs and task lists produced in the detailed planning stage make it possible to perform a thorough follow up of the dose account during the work. This is achieved through comparisons of the predicted and the received radiation doses. The VISIPLAN code only considers the transport of radiation through intervening shielding in the line of sight path from the source to the dose point. The photon fluence rate at a dose point originating from a volume source is determined by considering the volume source as consisting of a number of point sources. The photon fluence rate can be found by adding the contribution of every point source to the dose at the dose point. The photon fluency rate Φ (cm^−2^s^−1^) can be expressed as:

(1) $$\phi =\int v\frac{S.B.e^{-x}}{4\pi .\rho^{2}}dV$$

where S is the source strength representing the number of photons emitted by the source per unit volume and per unit time, B is the build-up factor, x represents the main free paths and ρ is the distance from a point source. This method is called “point kernel” integration. The volume integration scheme used in VISIPLAN is based on a Monte Carlo sampling of source positions in the source volume. The number of sampling points (Ns) can be chosen by the user. The point kernel equation above changes to the following form:

(2) $$\phi =\overset{N_{s}}{\underset{i=1}{\sum }}\frac{S_{tot}}{N_{S}}\;\frac{B.e^{-x_{i}}}{4.\pi .\rho_{i}^{2}}$$

where x_i_ and *ρ*_i_ are the mean free paths and the distance (cm) from the i-th sampling point, respectively. The equations above consider a monoenergetic photon source of source strength S_tot_. The sources encountered in many shielding problems emit photons at different energies. The VISIPLAN 3D ALARA planning tool uses, at present, a formulation where 25 energy bins are used. A source spectrum derived from other calculation codes needs to be re-grouped to the 25 energy group format when it is used in calculations with VISIPLAN. The photon fluence rate at the dose point in the energy group E_b_ is calculated as:

(3) $$\phi_{E_{b}}=\overset{N_{x}}{\underset{i=1}{\sum }}\frac{S_{tot}.F_{E_{b}}}{N_{s}}\frac{B_{E_{b}}}{4.\pi .\rho_{i}^{2}}$$

where F_Eb_ is the number of photons emitted in the energy group E_b_ per total activity S_tot_ of the source. The dose rate at the dose point is determined by using:

(4) $$Dose\;rate=\overset{25}{\underset{b=1}{\sum }}h_{Eb}.\phi_{E_{b}}$$

where *h_Eb_* is the dose conversion factor for energy *E_b_*. The dose rates in the VISIPLAN results are expressed in mSv.h^−1^. Input parameters, such as material, outer radius and length, were acquired from the designing parameters of Kori unit 1. The source terms used to perform the calculations during the decommissioning of the Kori unit-1 RPV are shown in Table 1 [14]. According to recent studies, the most significant radionuclide that contributed to worker doses in nuclear power plants is ^60^Co, which was responsible for over 80% of out-of-core radiation fields. 

Three working groups were considered for each of the three parts of the RPV. Each group had six cutters to carry out the operation of the fragmentation and segmentation of the RPV and one radiation protection officer (RPO) responsible for the safety of the cutters. The cutter was assumed to perform the cutting activities within the range of 30–38 cm, whereas the RPO was assumed to be located at a distance of 100–130 cm from the component. Four main commercial cutting technologies for decommissioning were introduced, i.e., waterjet cutting, laser cutting, shear cutting and plasma cutting technologies, as shown in Table 2. Waterjet cutting technology uses high pressure water for abrasive injection, whereas shear cutting uses two blades to cut an object on the same principle as a pair of scissors. Plasma cutting, which was selected as the best cutting technology for the reactor vessel in this study, uses a direct current arc to cause a metal oxidation reaction. 

## 3. Results and Discussion

**Figure 2:**
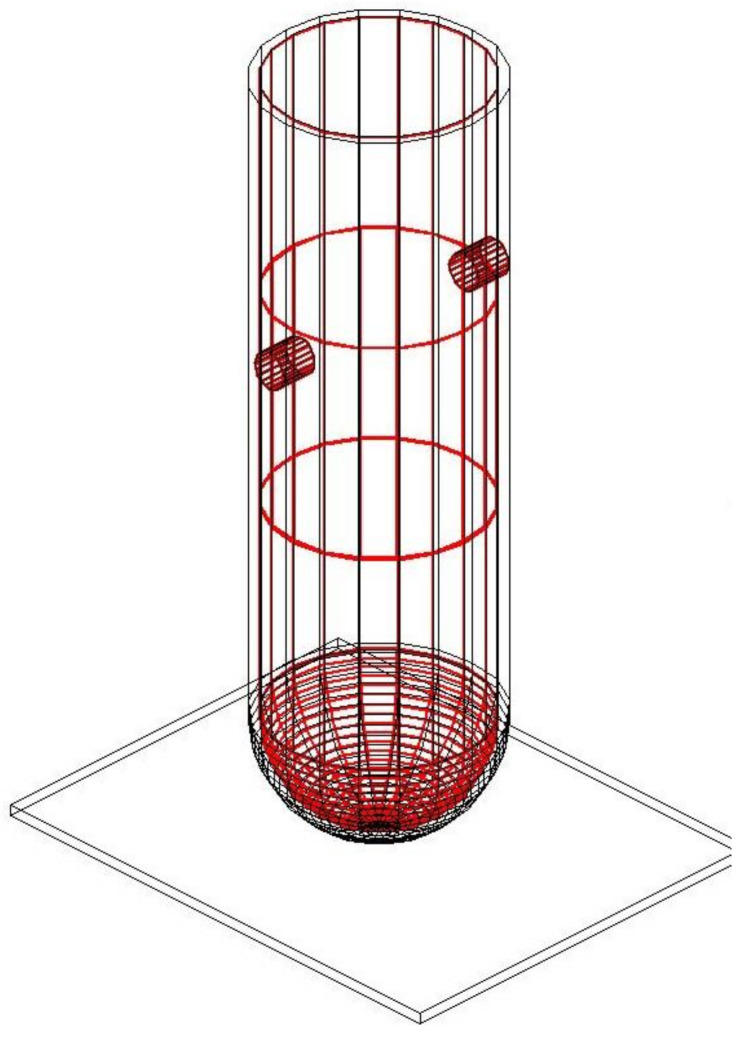
Illustration of the reactor pressure vessel at Kori unit 1.

**Figure 3:**
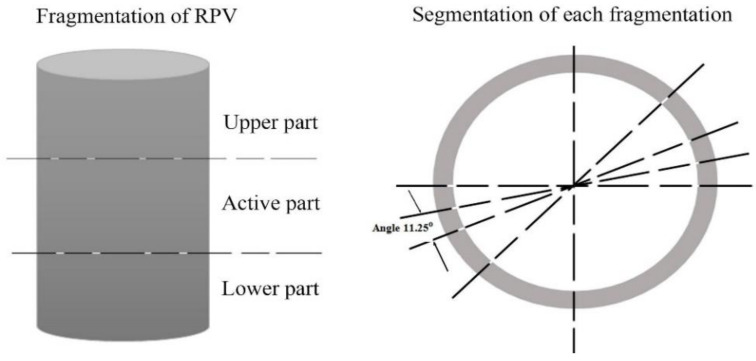
Description of reactor pressure vessel (RPV) and cylindrical cutting method.

The RPV, with a total height of 14.67 m, was divided into two separate parts, consisting of a cylindrical body and a spherical cap, as shown in Figure 2. The inside diameter of the RPV and the shell thickness was 4.17 m and 0.26 m, respectively, giving it a total diameter of 4.7 m. Based on the waste drum’s specification, the cylindrical body, with a length of 12.4 m, was first divided into 18 pieces using the fragmentation process and then each of the 18 pieces was cut to 32 segments. The spherical cap part, with a total length of 2.27 m, was divided into four pieces. The top two fragments were cut into 32 segments, while the remaining two smaller fragments were cut into 10 segments. In addition, six fragmentation operations were performed for the cold and hot legs, with a height of 0.68 m for each fragment, as shown in Figure 3. The total number of pieces for the whole cutting process was 664 pieces. This procedure for cutting the RPV was based on the dimensions of the waste drum, with a height and diameter of 0.8 m and 0.57 m, respectively. 

The cutting time for each axial part of the cylindrical body of the RPV was calculated as follows:

Circumference of RPV = L=2πr = 2 × 3.14 × 2.35 = 14.76 m

$$\mathrm{Time}=\frac{14760\;\mathrm{mm}}{15\;mm/min}=984\;\min$$

The cutting time for each axial part of the spherical cap of the RPV was calculated as follows:

Top two fragments’ circumference of the spherical cap of the RPV = 2 × 3.14 ×1.175 = 7.4 m

$$\mathrm{Time}=\frac{7400\;\mathrm{mm}}{15\;mm/min}=493\;\min$$

The two smaller fragments’ circumference of the spherical cap of the RPV = 2 × 3.14 × 0.587 = 3.69 m

$$\mathrm{Time}=\frac{3690\;\mathrm{mm}}{15\;mm/min}=246\;\min$$

The cutting time for each radial part of the RPV was calculated as follows:

Height of fragment = 0.68 m × 32 = 21.08 m

$$\mathrm{Time}=\frac{21080\;\mathrm{mm}}{15\;mm/min}=1405\;\min$$

The total time and dose prognoses for the fragmentation operation of the upper part of the RPV are presented in Table 3. Six cutters (1–6) were considered for the upper part fragmentation. The total time and accumulated dose for the cutting of each fragment per worker are shown in Table 4. The total work time taken for the RPO was 1896 minutes and the total accumulated dose was 1.0 × 10^2^ mSv. 

Cutters 7~12 were considered for one cutting of the active part of the RPV using the fragmentation process. For six active parts, the total time and accumulated dose are shown in Table 5. In Table 6, the total time and accumulated dose for the cutting of each fragment per worker are presented. For the radiation protection officer, the total work time taken was 948 min and the total accumulated dose was 2.8 × 10^3^ mSv.

Cutters 13–18 were considered for the operation of cutting the lower part. Table 7 shows the total time and accumulated dose for one cutting of the lower part of the RPV, whereas Table 8 shows the total time and accumulated dose for one cutting of each fragmentation per worker. The total work time for the radiation protection officer was 948 mins and the total accumulated dose was 1.10 × 10^2^ mSv. 

Using the segmentation process, cutters 1–6 were considered for cutting the upper part of the RPV. The total time and accumulated dose for the cutting of one segment of the upper part of the RPV is shown in Table 9. Table 10 presents the total time and accumulated dose for the cutting of each segment per worker. The total work time and total accumulated dose received by the the radiation protection officer was 1405 min and 1.60 × 10^2^ mSv, respectively. 

Cutters 7–12 were considered for the RPV active part segmentation. Table 11 shows the total time and accumulated dose for one segmentation process of the active part of the RPV. Table 12 shows the total time and accumulated dose for the cutting of each segment per worker. For the RPO, the total work time taken was 1405 min and the total accumulated dose was 1.00 × 10^3^ mSv. 

Table 13 presents the total time and accumulated dose for each segmentation process of the RPV for cutters 13–18. Table 14 presents the total time and accumulated dose for the cutting of each segment per worker. The time taken and total accumulated dose received by the radiation protection officer was 1405 minutes and 9.50 × 10^1^ mSv, respectively. 

Table 15 presents the collective time and dose to the decommissioning workers for both the fragmentation and segmentation operations. The total time duration, dose rate, task dose and accumulated dose are shown for the different cutter tasks. For the fragmentation process, the collective dose for the upper part, active part and lower part were 6.0 × 10^2^, 1.7 × 10^4^ and 9.1 × 10^2^ man-mSv, respectively. For the segmentation process, the collective dose for the upper part, active part and lower part were 5.7 × 10^2^, 6.2 × 10^3^ and 9.5 × 10^2^ man-mSv, respectively. The fragmentation of the active part contributed a higher dose compared to the other parts. The most significant radionuclide that contributed to the workers’ doses at Kori unit 1 was ^60^Co, which was responsible for over 80% of out-of-core radiation fields. Although ^55^Fe had the highest activities, the ^60^Co energy level was higher, hence the reason for its contribution. The time that was simulated for the fragmentation process of the upper, active and lower parts was 23.7 man-days, 23.7 man-days and 31.6 man-days, respectively. The time that was taken to cut the upper, active and lower parts using the segmentation process was 35.12 man-days, 35.12 man-days and 46.8 man-days, respectively. Segmentation operations took a longer time compared to the fragmentation process. Lastly, the total time that was taken to finish the fragmentation and segmentation processes of cutting the RPV of Kori unit 1 was 196 man-days.

**Figure 4:**
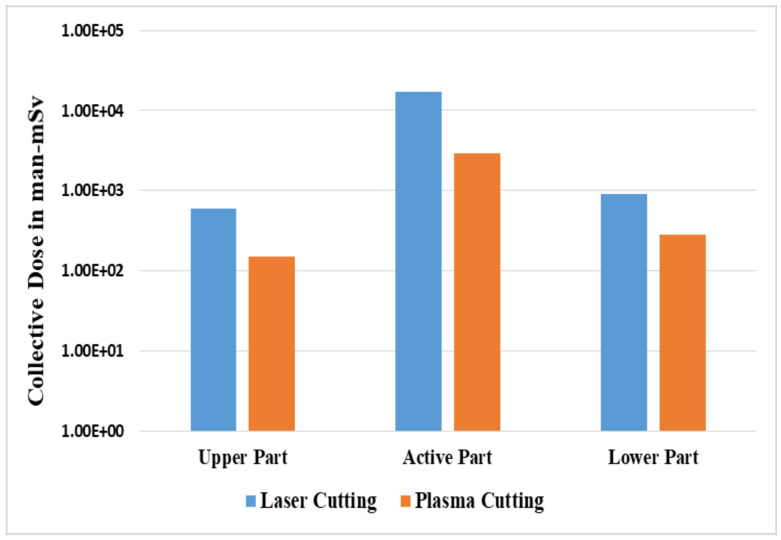
Comparison between laser cutting and plasma cutting for the fragmentation process.

**Figure 5:**
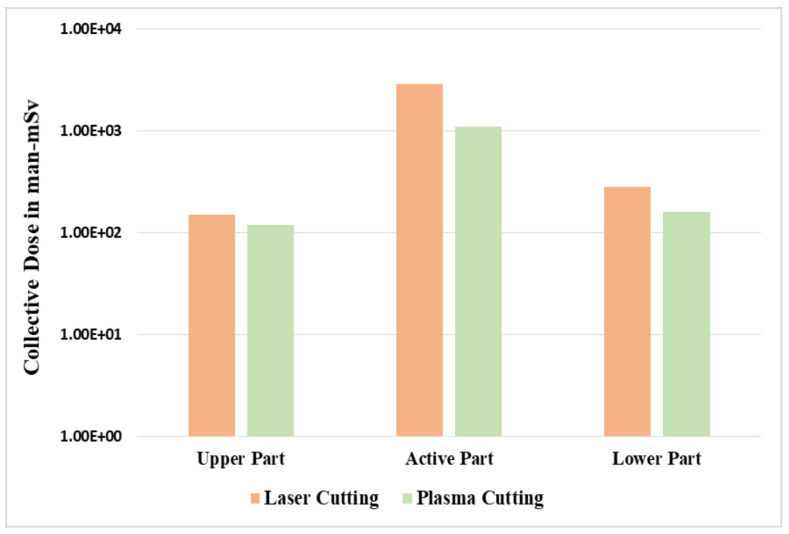
Comparison between laser cutting and plasma cutting for the segmentation process.

Sensitivity analysis was performed for the cutting technologies of laser and plasma for both the fragmentation and segmentation operations. The speed of plasma cutting was considered higher, at 15 mm/min, compared to 100 mm/min for the laser cutting, hence it took a longer time and led to a larger dose value received by the workers. The overall scenarios for laser cutting and plasma cutting are shown in Figure 4 and Figure 5. The occupational exposure of any worker should be controlled in order not to exceed the dose limits of 20 mSv per year averaged over five consecutive years, as recommended by the International Commission on Radiological Protection (ICRP). The values predicted in this study were relatively higher since we focused on the RPV, which was a primary component of the NPP, and thus strict measures, such as reduced time and increased shielding should be taken to protect decommissioning workers and keep the radiation exposure to the workers as low as possible. 

## 4. Conclusions

We performed the preliminary estimation of occupational exposure for radiation workers using the VISIPLAN 3D ALARA planning tool during the decommissioning phase of a reactor pressure vessel at Kori NPP unit 1, which will be scheduled in 2022–2028. Different cutting methods and cutting processes of fragmentation and segmentation of the reactor pressure vessel of Kori unit 1 were simulated in order to optimize the working time and exposure dose of radiation workers through quantitative risk modeling. It was found that VISIPLAN would be a good tool to plan and manage the occupational safety and health of radiation workers for the decommissioning of Kori NPP unit 1 in the near future.

## Figures and Tables

**Table 1:** Source terms in different parts of reactor pressure vessel.

Serial	Nuclide	Active Part (Bq)	Upper Part (Bq)	Lower Part (Bq)	Half-Life
1	^55^Fe	6.96 × 10^14^	1.28 × 10^13^	3.56 × 10^9^	2.74 years
2	^60^Co	2.23 × 10^12^	3.52 × 10^10^	5.25 × 10^6^	5.27 years
3	^152^Eu	6.62 × 10^9^	1.75 × 10^8^	1.56 × 10^4^	13.52 years
4	^154^Eu	1.23 × 10^9^	2.35 × 10^7^	1.65 × 10^3^	8.59 years
5	^134^Cs	2.60 × 10^8^	4.69 × 10^6^	9.08 × 10^2^	2.06 years
6	^155^Eu	3.76 × 10^7^	3.16 × 10^5^	1.50 × 10^1^	4.76 years
7	^110m^Ag	3.28 × 10^5^	3.75 × 10^3^	3.43 × 10^0^	250 days
8	^99^Tc	2.35 × 10^4^	3.96 × 10^2^	1.43 × 10^−2^	6 hours
**Total Activity**		6.98 × 10^14^	1.28 × 10^13^	3.59 × 10^9^	

**Table 2:** Thickness and speed of main cutting technologies.

	Water Jet Cutting	Laser Cutting	Shear Cutting	Plasma Cutting
Thickness	~20 cm	~20 cm	~20 cm	~20 cm
Speed	70–100 mm/min	15–45 mm/min	15–50 mm/min	150 mm/min

**Table 3:** Work time and estimated dose for the fragmentation of the upper part.

Total work time (min)	1896.0	Accumulated dose (mSv)	1.0 × 10^2^
Max. work time (min)	1968.1	Max. accumulated dose (mSv)	1.1 × 10^2^
Min. work time	1823.9	Min. accumulated dose (mSv)	9.4 × 10^1^

**Table 4:** Task information for the fragmentation of the upper part.

Task No.	Task Description	Duration (min)	Dose Rate (mSv/h)	Task Dose (mSv)	Accumulative Dose (mSv)
1	Cutter 1	135	1.30 × 10^1^	2.90 × 10^1^	2.90 × 10^1^
2	Cutter 2	165	8.30 × 10^−4^	2.30 × 10^−3^	2.90 × 10^1^
3	Cutter 3	129	7.50 × 10^0^	1.60 × 10^1^	4.50 × 10^1^
4	Cutter 4	172	7.00 × 10^0^	2.00 × 10^1^	6.50 × 10^1^
5	Cutter 5	165	7.60 × 10^−4^	2.10 × 10^−3^	6.50 × 10^1^
6	Cutter 6	182	1.10 × 10^1^	3.40 × 10^1^	1.00 × 10^2^
7	RPO 1	948	8.80 × 10^−4^	1.40 × 10^−2^	1.00 × 10^2^

**Table 5:** Work time and estimated dose for the fragmentation of the active part.

Total work time (min)	1896.0	Accumulated dose (mSv)	2.8 × 10^3^
Max. work time (min)	1971.0	Max. accumulated dose (mSv)	3.0 × 10^3^
Min. work time	1821.0	Min. accumulated dose (mSv)	2.6 × 10^3^

**Table 6:** Task information for the fragmentation of the active part.

Task No.	Task Description	Duration (min)	Dose Rate (mSv/h)	Task Dose (mSv)	Acc. Dose (mSv)
1	Cutter_7	165	5.70 ×10^2^	1.60 × 10^3^	1.60 × 10^3^
2	Cutter_8	172	3.10 × 10^1^	8.80 × 10^1^	1.60 × 10^3^
3	Cutter_9	182	2.10 × 10^1^	6.50 × 10^1^	1.70 × 10^3^
4	Cutter_10	165	3.20 × 10^2^	8.70 × 10^2^	2.60 × 10^3^
5	Cutter_11	129	1.90 × 10^1^	4.10 × 10^1^	2.60 × 10^3^
6	Cutter_12	135	8.10 × 10^1^	1.80 × 10^2^	2.80 × 10^3^
7	RPO 2	948	2.60 × 10^−2^	4.10 × 10^−1^	2.80 × 10^3^

**Table 7:** Work time and estimated dose for the fragmentation of the lower part.

Total work time (min)	1896.0	Accumulated dose (mSv)	1.1 × 10^2^
Max. work time (min)	1964.5	Max. accumulated dose (mSv)	1.2 × 10^2^
Min. work time	1827.5	Min. accumulated dose (mSv)	1.1 × 10^2^

**Table 8:** Task information for the fragmentation of the lower part.

Task No.	Task Description	Duration (min)	Dose Rate (mSv/h)	Task Dose (mSv)	Acc. Dose (mSv)
1	Cutter_13	129	1.20 × 10^1^	2.70 × 10^1^	2.70 × 10^1^
2	Cutter_14	165	1.20 × 10^−2^	3.30 × 10^−2^	2.70 × 10^1^
3	Cutter_15	135	7.80 × 10^0^	1.80 × 10^1^	4.40 × 10^1^
4	Cutter_16	182	9.30 × 10^0^	2.80 × 10^1^	7.20 × 10^1^
5	Cutter_17	165	7.00 × 10^−3^	1.90 × 10^−2^	7.20 × 10^1^
6	Cutter_18	172	1.40 × 10^1^	4.10 × 10^1^	1.10 × 10^2^
7	RPO_3	948	1.30 × 10^−2^	2.10 × 10^−1^	1.10 × 10^2^

**Table 9:** Work time and estimated dose for the segmentation of the upper part.

Time and Dose Prognoses			
Total work time (min)	2810.0	Accumulated dose (mSv)	1.6 × 10^2^
Max. work time (min)	2826.3	Max. accumulated dose (mSv)	1.6 × 10^2^
Min. work time	2793.9	Min. accumulated dose (mSv)	1.6 × 10^1^

**Table 10:** Task information for the segmentation of upper part.

Task No.	Task Description	Duration (min)	Dose Rate (mSv/h)	Task Dose (mSv)	Acc. Dose (mSv)
1	cutter_1	220	3.80 × 10^0^	1.40 × 10^1^	1.40 × 10^1^
2	cutter_2	300	4.20 × 10^0^	2.10 × 10^1^	3.50 ×10^1^
3	cutter_3	260	8.90 × 10^0^	3.90 × 10^1^	7.30 × 10^1^
4	cutter_4	200	8.40 × 10^0^	2.80 × 10^1^	1.00 × 10^2^
5	cutter_5	185	4.40 × 10^0^	1.30 × 10^1^	1.10 × 10^2^
6	cutter_6	240	2.70 × 10^0^	1.10 × 10^1^	1.30 × 10^2^
7	RPO	1405	1.40 × 10^0^	3.30 × 10^1^	1.60 × 10^2^

**Table 11:** Work time and estimated dose for the segmentation of the active part.

Total work time (min)	2810.0	Accumulated dose (mSv)	1.0 × 10^3^
Max. work time (min)	2836.8	Max. accumulated dose (mSv)	1.0 × 10^3^
Min. work time	2783.2	Min. accumulated dose (mSv)	1.0 × 10^3^

**Table 12:** Task information for the segmentation of the active part.

Task No.	Task Description	Duration (min)	Dose Rate (mSv/h)	Task Dose (mSv)	Acc. Dose (mSv)
1	cutter_1	260	3.60 × 10^1^	1.60 × 10^2^	1.60 × 10^2^
2	cutter_2	200	3.50 × 10^1^	1.20 × 10^2^	2.70 × 10^2^
3	cutter_3	220	2.70 × 10^1^	9.90 × 10^1^	3.70 × 10^2^
4	cutter_4	185	4.10 × 10^1^	1.30 × 10^2^	5.00 × 10^2^
5	cutter_5	300	3.60 × 10^1^	1.80 × 10^2^	6.80 × 10^2^
6	cutter_6	240	3.60 × 10^1^	1.40 × 10^2^	8.30 × 10^2^
7	RPO 1	1405	9.20 × 10^0^	2.10 × 10^2^	1.00 × 10^3^

**Table 13:** Work time and estimated dose for the segmentation of the lower part.

Total work time (min)	2810.0	Accumulated dose (mSv)	9.5 × 10^1^
Max. work time (min)	2838.0	Max. accumulated dose (mSv)	9.6 × 10^1^
Min. work time	2782.0	Min. accumulated dose (mSv)	9.4 × 10^1^

**Table 14:** Task information for the segmentation of the lower part.

Task No.	Task Description	Duration (min)	Dose Rate (mSv/h)	Task Dose (mSv)	Acc. Dose (mSv)
1	Cutter_1	185	4.20 × 10^0^	1.30 × 10^1^	1.30 × 10^1^
2	Cutter_2	260	8.00 × 10^0^	3.40 × 10^1^	4.70 × 10^1^
3	Cutter_3	220	4.00 × 10^0^	1.50 × 10^1^	6.20 × 10^1^
4	Cutter_4	200	2.10 × 10^0^	6.90 × 10^0^	6.90 × 10^1^
5	Cutter_5	240	1.50 × 10^0^	6.20 × 10^0^	7.50 × 10^1^
6	Cutter_6	300	1.40 × 10^0^	6.80 × 10^0^	8.20 × 10^1^
7	RPO	1405	5.60 x 10^−1^	1.30 x 10^1^	9.50 x 10^1^

**Table 15:** Collective time and dose of fragmentation and segmentation processes.

**Scenario**	**Collective Time (man-h)**	**Collective Dose (man-mSv)**
Upper part fragmentation	189.60	6.0 × 10^2^
Active part fragmentation	189.60	1.7 × 10^4^
Lower part fragmentation	252.80	9.1 × 10^2^
**Scenario**	**Collective Time (man-h)**	**Collective Dose (man-mSv)**
Upper part segmentation	281.00	5.7 × 10^2^
Active part segmentation	281.00	6.2 × 10^3^
Lower part segmentation	374.60	9.5 × 10^2^
Total	1568.6 man-h = 196 man-days	
